# Supplementary material for: A Humanized Yeast Phenomic Model of Deoxycytidine Kinase to Predict Genetic Buffering of Nucleoside Analog Cytotoxicity
Source: Genes (Basel). 2019 Sep 30;10(10):770. doi: 10.3390/genes10100770 (PMC6826991; doi:10.3390/genes10100770)
Supplement: Supplementary file 1 [file genes-10-00770-s001.zip › Additional_Files/Additional_File6_GTA.zip!/Additional_File6_GTA/B - GTA_L.html]

plotly
